# Antisocial personality disorder and therapeutic pessimism – how can mentalization-based treatment contribute to an increased therapeutic optimism among health professionals?

**DOI:** 10.3389/fpsyg.2024.1320405

**Published:** 2024-02-21

**Authors:** Emilie Flaaten, Maria Langfeldt, Katharina T. E. Morken

**Affiliations:** ^1^Drammen Hospital, Outpatient Team for Addiction and Mental Health, Vestre Viken Hospital Trust, Drammen, Norway; ^2^Blakstad Hospital, Section of Security Psychiatry, Vestre Viken Hospital Trust, Drammen, Norway; ^3^Department of Addiction Medicine, Haukeland University Hospital, Bergen, Norway; ^4^Department of Clinical Psychology, University of Bergen, Bergen, Norway

**Keywords:** antisocial personality disorder, therapeutic pessimism, mentalization, mentalization-based treatment, treatability, specialized psychotherapy

## Abstract

Antisocial personality disorder (ASPD) is associated with therapeutic pessimism among health professionals. Several variables are associated with obstacles in therapist’s willingness to treat ASPD. Variables that are relevant are (i) confusion associated with the term ASPD, (ii) characteristics of the disorder, (iii) attitudes, experiences, and knowledge clinicians possess, and (iv) insufficient management of countertransference. We assume that therapeutic pessimism is related to the lack of evidence-based, effective treatment for individuals with ASPD. This is problematic because ASPD is associated with large socio-economic costs and considerable suffering for the individual and the society. Mentalization-based treatment (MBT) was developed in treating borderline personality disorder (BPD) and is now considered an effective treatment for this group. Mentalization is defined as the process by which individuals make sense of themselves and others in terms of subjective states and mental processes. This ability affects an individual’s psychological functioning, mental health, self-organization, and interpersonal relationships. The overall goal of MBT is to strengthen the individual’s mentalizing abilities and facilitate more adaptive handling of problematic, internal states. Recently, a version of MBT tailored for individuals with ASPD (MBT-ASPD) has been developed. The purpose of this review is to investigate how MBT-ASPD relates to the major obstacles that contribute to the therapeutic pessimism toward this group. Despite a limited evidence base, preliminary studies indicate promising results for MBT-ASPD. More research is still required, this review suggests MBT-ASPD can contribute to increased therapeutic optimism and demonstrate specific characteristics of MBT-ASPD that contribute to management of therapeutic pessimism.

## Introduction

In their clinical guidelines, the National Institute for Care and Excellence (NICE) points out how the “therapeutic pessimism” among health professionals toward antisocial personality disorder (ASPD) seems to be repeatedly highlighted in the literature (National Institute for Health and Care Excellence, 2010). A stereotypical perception of ASPD is widespread (Bateman et al., 2013; van Dam et al., 2022). The negative attitudes toward people with ASPD have been characterized by the notion that this group is untreatable or could even worsen from treatment (Rice et al., 1992). In recent years, this opinion appears to have changed somewhat in line with increased focus and research on the potential treatment opportunities for ASPD. However, at present there is still no evidence-based treatment for this group (National Institute for Health and Care Excellence, 2010; Gibbon et al., 2020) Understandably, with a lack of effective treatment options for ASPD, therapeutic pessimism among health professionals will likely persist.

Several attempts have been made to ensure that ASPD is no longer a diagnosis of exclusion (Pickersgill, 2009). NICE has developed clinical guidelines that cover general and specific treatment principles for ASPD, as well as proposals for potential preventive measures and early intervention. Two Cochrane reviews with a 10-year gap have investigated several psychological interventions for ASPD (Gibbon et al., 2010, 2020). The latest review summarizes the findings from 19 randomized controlled trials (RCTs) comparing 18 different psychotherapies to treatment as usual (TAU). Some RCTs indicate that specialized psychotherapy may be more effective than TAU for individuals with ASPD. However, both Cochrane reviews concluded that there is a lack of high-quality evidence on how people diagnosed with ASPD can be treated effectively (Gibbon et al., 2010, 2020). In a recent published RCT, the researchers compared Schema Therapy (ST) to TAU in offenders with personality disorders and aggression. They found that ST produced more rapid improvements than TAU (Bernstein et al., 2023). Although the findings need replication, the study contradicted the belief that people with ASPD are untreatable. Summed up, NICE guidelines summarize several therapeutic principles that may be useful when treating individuals with ASPD and findings from several RCTs show that specialized psychotherapy may be more effective than TAU. However, there is still an insufficient treatment provision for this patient group (National Institute for Health and Care Excellence, 2010).

For a long time, borderline personality disorder (BPD) was regarded as an untreatable disorder (Chanen et al., 2007; McMain et al., 2009; Choi-Kain et al., 2017). This therapeutic pessimism undoubtedly contributed to the stigmatization experienced by those who received a BPD diagnosis. It seemed that many believed that the defining characteristic of a personality disorder is that the patient’s patterns of social dysfunction are enduring (Bateman and Fonagy, 2016, p. 435). Nonetheless, there are several effective therapies for BPD today. In the latest Cochrane review (2020), including 75 RCTs, different types of psychotherapies were compared to TAU or no treatment at all. At least six BPD-tailored treatments appear more effective than TAU (Storebø et al., 2020). This has contributed to the notion that patients with BPD should no longer be regarded as untreatable. Consequently, therapeutic pessimism among health professionals has significantly decreased (Bateman and Fonagy, 2016, p. 435).

Mentalization-based treatment (MBT) is one of the treatments found to be effective for BPD (Storebø et al., 2020). RCTs with follow-up studies have found that MBT for BPD can reduce suicidal and self- injurious behaviors, improve interpersonal functioning, and reduce the number of hospitalizations compared to TAU (Bateman and Fonagy, 1999, 2008b). It has recently been proposed that people diagnosed with ASPD could also benefit from MBT, given that this patient group struggles with mentalizing too (Bateman et al., 2016). The aim of MBT is to increase the mentalization capacities of patients. The original framework of the model has therefore been modified to improve outcomes for individuals with ASPD and the adaptation has been named MBT-ASPD (Bateman and Fonagy, 2016, p. 74). Smaller studies have shown that MBT could effectively reduce aggression in patients with ASPD (McGauley et al., 2011) and for patients with comorbid ASPD and BPD (Bateman et al., 2016). Currently, there is an ongoing multicenter RCT in London investigating MBT-ASPD’s effectiveness for male offenders compared to probation as usual (Fonagy et al., 2020). MBT has also developed a program for youth with conduct disorder (MBT-CD), promising results have been published from the feasibility study (Hauschild et al., 2023) and there is an ongoing RCT on MBT-CD in Germany (Taubner et al., 2021).

The current research on how to effectively treat ASPD remains lacking, and, within the provision of TAU, health professionals appear to be pessimistic about the treatment opportunities for individuals diagnosed with ASPD. One study on therapist motivation for working clinically with ASPD found that only 12% were motivated to work with this patient group (van Dam et al., 2022). Therapeutic pessimism is likely correlated with less effective treatment outcomes for individuals with ASPD. The aim of this review is to investigate how one specific treatment program, MBT-ASPD, solve the obstacles in the willingness to treat ASPD among health professionals.

### Why is treatment pessimism surrounding ASPD?

ASPD has been treated for years, in forensic and regular mental health settings (Gibbon et al., 2020). There are treatment options offered to individuals with ASPD, but they appear to fall short to effectively treat the disorder. The unmet needs of many individuals with ASPD reflect a need for a change in the health services currently provided (National Institute for Health and Care Excellence, 2010). It appears that the therapeutic pessimism among health professionals may be an especially important reason for the lack of effective treatment options for ASPD. Reviewing the most updated guidelines for ASPD (National Institute for Health and Care Excellence, 2010), four main themes associated with therapeutic pessimism are particularly repeated. These are: (i) confusion related to psychopathy and ASPD, (ii) treatment rejecting behavior, (iii) refused rather than accepted into treatment and (iv) inadequate management of countertransference. An investigation of these obstacles could be an important step toward finding effective treatments for individuals with ASPD.

### ASPD and psychopathy – two terms creating confusion

ASPD and psychopathy are terms that have been debated and understood differently throughout the history. Various debates on what ASPD and psychopathy is, and a growing interest for more clinical psychological perspectives, has shaped the development of the diagnosis and its nosology. Originally in the context of DSM II and DSM III, scholars argued for the existence of two different personality types: one more callous with low anxiety, a so-called psychopathic subtype, and one more erratically and affectively driven aggressive personality, the sociopathic or antisocial subtype (Pickersgill, 2012). Today there is some confusion as to what one means when discussing this diagnosis. The DSM-IV and 5 version of ASPD contains more behavioral traits like impulsivity, deceitfulness, recklessness, aggressiveness, unlawful behaviors, and irresponsibility and fewer psychopathic personality traits. Hence the ASPD DSM is more inclusive than for instance the psychopathic personality as defined by PCL-R (Hare, 2003) which covers more of the intrapsychic aspects of the disorder such as callousness, grandiosity, shallow affect, glibness and manipulativeness together with overlapping behavioral traits similar to the ASPD diagnosis in the DSM. Thus, even though ASPD and psychopathy share some characteristics, there are differences between the two conditions. ASPD is a broader more inclusive term that is largely based on behavioral traits such as antisocial behavior and criminality.

However, ASPD and psychopathy are sometimes understood as synonymous terms (Ogloff et al., 2016), which contribute to added confusion. For instance, in the DSM-5, the introduction of ASPD includes “this pattern has also been referred to as psychopathy” (American Psychiatric Association, 2013, p. 659). The DSM-5 criteria for ASPD include behavioral traits such as failure to obey laws and norms, lying and deception, impulsive behavior, irritability and aggression, disregard of safety of self and others, irresponsibility, and lack of remorse for actions. Three of these criteria, inclusive age above at least 18 and a presence of conduct disorder in childhood must be present for the diagnosis to be set (DSM-5). Psychopathy, on the other hand, is more heavily based on fundamental personality deficits (Ogloff and Wood, 2010). Psychopathy is characterized by a behavioral pattern of impulsivity, shallow affect, superficial charm, callousness, and manipulation that deviates from the social, moral, or legal norms of society (Werner et al., 2015; Sanz-García et al., 2021). Further, only ASPD is a clinical diagnosis incorporated in the DSM-5. Psychopathy is not regarded as a formal diagnosis, but rather as a severe type of personality disorder that combines both narcissistic traits and ASPD traits in addition to more severe personality deficits. In the triarchic psychopathy model by Patrick and Drislane (2015) boldness has been suggested as a primary difference in personality traits between ASPD and psychopathy, boldness is a trait characterized by fearlessness, dominance, and low stress reactivity (Wall et al., 2015).

Psychopathy can be measured with different assessment tools, including the Psychopathy Checklist-Revised (PCL-R) (Hare, 2003). Research has found that most individuals with psychopathy fulfill the diagnostic criteria for ASPD (Ogloff et al., 2016), but only 25–30% of individuals with ASPD have significant psychopathic traits (Hare and Neumann, 2008; Bo and Kongerslev, 2016). This suggests that ASPD and psychopathy represent two different disorders. This is, however, not a unified opinion within the field. Some researchers have proposed that psychopathy and ASPD may underline the same natural class (Skilling et al., 2002); capture the same disorder (Widiger, 2006); or even that psychopathy can be considered a severe form of ASPD (Coid and Ullrich, 2010). The distinguishment between primary and secondary psychopathy, where primary psychopathy is characterized by low stress reaction and social dominance and secondary psychopathy is characterized by high levels of aggression and more impulsivity, is by some seen as the distinguishment between ASPD (secondary psychopathy) and psychopathy (primary psychopathy) (Wall et al., 2015).

Lately, the dimensional perspective on personality disorder suggests that perhaps it could be wise to view psychopathy and antisocial PD as phenomena on the same continuum. In the alternative model of DSM-5, the workgroup made an effort to include more of the self and other functioning deficits in the diagnosis of ASPD, thus becoming more aligned with the psychopathy definition of the PCL-R (Mark et al., 2022). The dimensional diagnosis of ASPD has a psychopathy specifier where three traits need to be fulfilled in addition to the general ASPD criteria, these are low levels of anxiousness, withdrawal and attention seeking. In the dimensional model in ICD-11 and DSM-5, ASPD is found in a combination of the trait domain of antagonism and disinhibition (Mulder et al., 2021; Mark et al., 2022). However, there are still authors who question the dimensional aspect of ASPD and psychopathy and continue to stress that those are two different conditions (Mark et al., 2022). Fonagy and Luyten (2018) has suggested different pathways linking mentalizing with aggression. And the newer developments on conduct disorder and MBT, for instance in a feasibility study on MBT for conduct disorder (MBT-CD), suggest that psychopathy can be understood in a continuum from conduct disorder. They do not differentiate between psychopathic and antisocial traits (Hauschild et al., 2023). The disagreement on whether psychopathy and ASPD are dimensions of the same disorder or different conditions is of clinical interest. Psychopathy as a phenomenon is related to several misconceptions and myths among health professionals (Berg et al., 2013).

However, it is likely that psychopathic traits complicate treatment to a greater extent. This includes characteristics such as callousness and fearlessness, which can hinder the development of a therapeutic alliance, as well as behavioral patterns that disrupt the utility of group therapy. These characteristics are usually less prominent in individuals with ASPD without psychopathy (Frick and White, 2008; Wall et al., 2015). This could imply a need for different treatment options for the two groups. De Visser et al. (2023) has given some nuanced perspectives on how to tailor psychotherapeutic treatment better for ASPD patients with various levels of severity and deficiencies in social cognition, including those with psychopathic features. De Visser suggests that for ASPD with psychopathic features specific treatment approaches are recommended, like cognitive remediation training or risk reduction approaches. For other types of ASPD, with attachment related disturbances and mistrust as core problem, trauma interventions are recommended. Furthermore, the ASPD group that have trouble with mentalizing other, or reciprocity in relation to understanding others from within, would benefit from treatments like MBT. Therefore, using ASPD and psychopathy synonymously can potentially have harmful effects and prevent appropriate treatment for both disorders (Ogloff et al., 2016).

### Treatment-rejecting rather than treatment-seeking

Repeated rule- breaking, recklessness, deceitfulness, impulsiveness, aggression, and irresponsibility are characteristics that distinguish ASPD from other mental illnesses; therefore, there is no surprise that many individuals with ASPD do not take on the traditional patient role (McGauley et al., 2011). The foundation of a therapeutic alliance is perhaps the most investigated and essential element leading to change and success in all psychotherapies and refers to the collaborative work of the therapist-patient relationship (Bordin, 1979; Flückiger et al., 2018). Because of the characteristics of ASPD, establishing a working alliance could be challenging.

One reason seems to be that many individuals with ASPD do not recognize their personality problems and rarely shows up voluntarily to treatment. This behavior is referred to as being treatment-rejecting rather than treatment- seeking (Tyrer et al., 2003; National Institute for Health and Care Excellence, 2010). Treatment-rejecting individuals are often unwilling to recognize their core personality problems and less motivated to change (Tyrer et al., 2003). Typically, individuals with ASPD tend to seek mental health services for other reasons than ASPD, for example for co-existing mental disorders (McGauley et al., 2011). Further, many individuals with ASPD are also coerced into therapy, for example by a relative or an authority. For that reason, engaging individuals with ASPD in therapy for personality issues is challenging (National Institute for Health and Care Excellence, 2010). Moreover, the drop-out rate for individuals with ASPD in therapy is high (Huband and Duggan, 2007; McGauley et al., 2011).

Building a bond with the patient is also a challenging task for the therapist. Individuals with ASPD have a particular sensitivity to hierarchy and power, which could impede the development of a therapeutic relationship. Individuals with ASPD may look at the clinician as an authority. Often, they tend to challenge perceived or actual authorities because their interpersonal relationships are based on competition, and domination (Bateman and Fonagy, 2016, p. 398). Therefore, boundary violation in therapy is common (McGauley et al., 2011). Another aspect to consider is whether the patient is actually cooperating and making progress. Illusory treatment alliance is a term used to describe an erroneous perception that a therapeutic alliance exists. According to psychoanalytic literature, these perceptions often stem from the therapists’ own wishful projections (Meloy and Yakeley, 2014, p. 1023). There is a risk that some patients with ASPD may display an illusory treatment alliance to gain certain advantages. For example, the patient could exhibit an apparently enhanced self-understanding in order to manipulate the therapist to recommend certain privileges (Bender, 2005), such as earlier parole from prison or increased child visitation. Consequently, the therapist is encouraged to engage the environment around individuals with ASPD in therapy (National Institute for Health and Care Excellence, 2010). DSM-5 has emphasized this issue in its diagnostic manual: “Because deceit and manipulation are central features of antisocial personality disorder, it may be especially helpful to integrate information acquired from systematic clinical assessments with information collected from collateral sources” (American Psychiatric Association, 2013, p. 659).

It appears to be particularly difficult to establish a good working alliance with individuals with ASPD, primarily because of treatment-rejecting behavior and characteristics that affect the therapeutic relationship negatively. However, recent studies have identified factors related to establishing a positive therapeutic alliance such as tailoring the treatment, being attentive, authentic and non-judgmental, together with maintaining a firm stance or uphold boundaries (Morken et al., 2022; Aerts et al., 2023).

### Refused rather than accepted into treatment

A third possible explanation for why it has been shown difficult to find effective treatment options for individuals with ASPD could be characteristics in the hands of the service providers. While many individuals with ASPD are treatment-rejecting (Tyrer et al., 2003), many are also rejected by service providers. In fact, just being diagnosed with ASPD could be used as an exclusion criterion to get admission into mental healthcare (van Dam et al., 2022). In a study conducted by Eren and Şahin (2016), they investigated mental health professionals’ (*n* = 332) attitudes toward individuals with a personality disorder. They found that the mental health professionals considered ASPD difficult to treat, compared to other personality disorders, and had negative attitudes toward them (Eren and Şahin, 2016). Besides, research shows that generally individuals with personality disorders are considered more difficult to treat. Therefore, ASPD as a label could negatively affect clinicians’ attitudes toward treatment outcomes (Beryl and Völlm, 2018). In addition to the question of “treatability” regarding ASPD, the willingness to work with this patient group appears to play an important role.

In another study, the researchers studied a group of clinicians (*n* = 130) working in regular and forensic mental health services, and their willingness to work with individuals with ASPD (van Dam et al., 2022). The results showed that approximately 60% of the clinicians working in regular mental health services indicated that they were not motivated to work with ASPD patients. 28% of the participants reported that they were ambivalent, whereas only 12% stated that they were motivated to work with this patient group. Among the clinicians working in forensic mental health settings, the willingness was higher (65%). However, approximately one-third of the clinicians in forensic mental health services expressed ambivalence or unwillingness to work with patients with ASPD. The researchers found that the clinicians who worked with individuals diagnosed with ASPD generally had more positive feelings toward ASPD patients compared to those who did not. There could be several reasons for this, for example, that the clinicians working with individuals with ASPD are initially more motivated to work in this field. It could also be that working with individuals with ASPD generates positive feelings due to experienced improvement in therapy (van Dam et al., 2022).

The final factor, perceived behavioral control, appears to be the greatest predictor of willingness to work with this group. The researchers point out how important it is that training leads to a sense of mastery. To achieve this, the researchers emphasize the importance of having clear manuals and instructions combined with training and supervision (van Dam et al., 2022). These findings are also supported by results in previous studies, indicating that the level of education, expertise, and clinical supervision of mental health professionals affect perceived difficulties and attitudes toward people with personality disorders (Eren and Şahin, 2016).

To summarize, studies show that the lack of experience and knowledge about ASPD affects certain attitudes among health professionals. The studies highlight the importance of gaining experience, sufficient training, and supervision in the treatment of ASPD, and that negative attitudes from clinicians can be damaging, increase stigma and reduce the willingness to work with individuals with ASPD (Eren and Şahin, 2016; Beryl and Völlm, 2018; van Dam et al., 2022).

### Countertransference in psychotherapeutic settings

Countertransference represents another subject that can complicate the psychological treatment of individuals with ASPD. Countertransference refers to the therapist’s various conscious and unconscious reactions, e.g., emotions, toward their patients that occur during the therapy sessions (American Psychological Association, n.d.-a). It is considered a fundamental component in all psychotherapies (Colli and Ferri, 2015; Lingiardi et al., 2015), which can influence psychotherapy outcomes both positively and negatively (Hayes et al., 2018). According to Colli and Ferri (2015), “the therapist’s emotional responses [countertransference] to patients can inform the clinician about the patient’s personality, impact the therapy outcome, influence patient resistance and elaboration, mediate the influence of therapist interventions and influence therapeutic alliance and alliance ruptures’ resolution.”

In an article from Hayes et al. (2018) summarizing findings from three meta-analyses, they investigated how countertransference reactions in therapists are related to psychotherapy outcomes. They found that better countertransference management is associated with better treatment outcomes. Higher levels of self-insight, conceptualizing capacities, empathy, self-integration abilities, and anxiety management are considered features that can facilitate better management of countertransference reactions (Hayes et al., 2018). Thus, it is rather how the therapist relates to the concept, not countertransference itself, that is crucial for how countertransference reactions affect therapy.

Countertransference can potentially be a source for more complex knowledge about a patient from a therapist’s point of view (Karterud et al., 2020, p. 172), and facilitate a better understanding of what is going on in the patient’s inner world and in the therapeutic interaction (Karterud et al., 2020, p. 173). As a consequence, countertransference may serve as a useful, productive tool in therapy as long as therapists learn how to identify, be aware of, and manage countertransference reactions that arise in therapy. However, this may be challenging. Therefore, countertransference can hinder effective treatment of different mental health issues (Fauth, 2006; Harrison and Westwood, 2009; Hofsess and Tracey, 2010), such as ASPD (Stefana et al., 2020).

Although countertransference can affect outcomes of all psychotherapies, it is found to be particularly important in the treatment of personality disorders (Colli and Ferri, 2015). Patients with ASPD typically exhibit a behavioral pattern characterized by manipulation, lack of trust, cooperation difficulties, non-compliance, and defensiveness over the course of treatment (McWilliams, 1994). Additionally, they tend to struggle to deal with challenging things internally; therefore, they tend to project their internal world and problems onto their surroundings instead. This typically evokes particular reactions in therapists (Ruszczynski, 2010), more specifically shock, hostility, moralistic outrage, hatred, fear, dread, and feelings of helplessness. These tendencies have been shown to be independent of the treatment setting and the therapist’s theoretical orientation (Schwartz et al., 2007).

Stefana et al. (2020) conducted a systematic review of the relationship between patients’ personality pathology and psychotherapists’ emotional, cognitive, and behavioral reaction patterns in individual psychotherapy settings. The researchers found that studies indicate that antisocial personality traits are positively correlated with feelings of being mistreated, criticized, and devalued (Colli et al., 2014; Tanzilli et al., 2016), as well as annoyed and angry (Tanzilli et al., 2016). Additionally, emotionally dysregulated patients, such as patients with ASPD, tend to evoke a greater portion of anxiety and feelings of incompetence among clinicians (Stefana et al., 2020). In summary, negative countertransference reactions appear to be prevalent among therapists working with individuals with ASPD.

**Figure 1:**
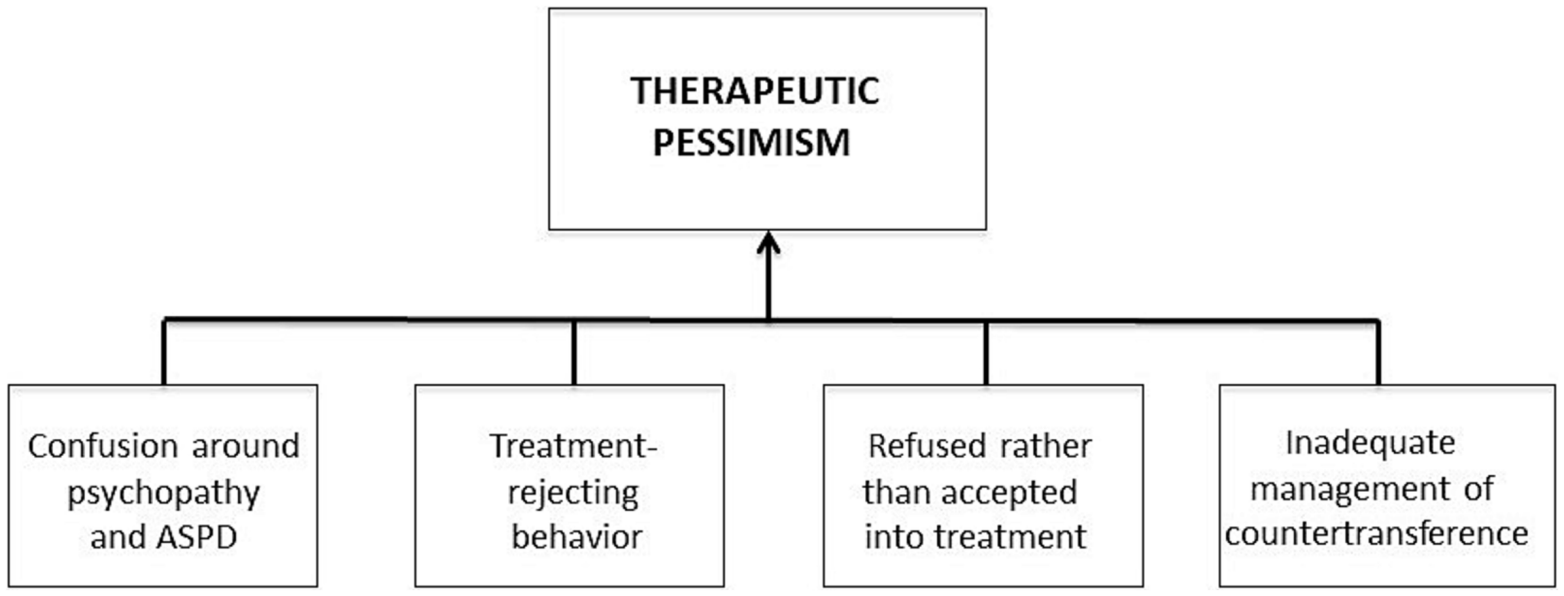
A summary of the therapeutic pessimism toward individuals with ASPD among health professionals.

In conclusion, as demonstrated in Figure 1, insufficient management of negative countertransference reactions combined with confusion around ASPD and psychopathy, treatment-rejecting behavior, and refusing patients from treatment, appear to be the main variables underlying the therapeutic pessimism toward individuals with ASPD among health professionals.

### Mentalization-based treatment for ASPD

MBT is a treatment rooted in psychoanalytic, attachment, and social cognition theories that highlights mentalization as crucial for an individual’s psychological functioning and mental health (Choi-Kain and Unruh, 2016). Mentalizing refers to “the process by which we make sense of each other and ourselves, implicitly and explicitly, in terms of subjective states and mental processes” (Bateman and Fonagy, 2010). Mentalizing is important for various aspects of an individual’s life, such as self-organization, affect regulation, interpersonal communication, and social relations (Bateman and Fonagy, 2016, p. 3). Good mentalizing can manifest in different ways, such as exhibiting a curious, not-knowing, and genuine interest in one’s own and others’ thoughts and feelings. It can also include accepting that people have different perspectives or being open and able to forgive other people based on an understanding that their mental state affects behavior (Bateman and Fonagy, 2016, p. 117).

A developmental perspective is closely related to how MBT links mentalizing to the development of personality disorders. The mentalizing capacity develops in the context of interactions with others, and its quality in relation to understanding others is influenced by how well those around the individual mentalize. Therefore, children who grow up in secure households are more likely to develop secure attachment styles and in turn enhanced mentalization processes. Conversely, children who grow up in insecure households and experience significant trauma, will be unable to feel safe about what others think of them. This can lead to a poorly developed capacity to mentalize, deficits in empathy, and difficulty distinguishing one’s own mental states from others’ (Yakeley, 2014).

New empirical evidence supports the hypothesis that ASPD is a developmental disorder rooted in insecure attachment (Yakeley, 2014). Bateman and Fonagy (2008a) propose that a significant proportion of individuals who meet the criteria for ASPD have experienced trauma and disruptions to their attachment system in childhood. In a study investigating the association between ASPD, childhood trauma history, and dissociative symptoms, male patients diagnosed with ASPD (*n* = 579) were compared to a healthy control group (*n* = 599). 60.8% of the individuals with ASPD compared to 37.4% of the controls had experienced at least one type of traumatic event. Besides, individuals with ASPD had significantly higher rates of childhood physical and sexual abuse, neglect, as well as early separation. The authors concluded that childhood trauma plays a key role in the development of antisocial behavior (Semiz et al., 2007).

The MBT framework suggests that such experiences deactivate the attachment system and disrupt mentalizing capacities of individuals with ASPD. Individuals with ASPD struggle to understand their own inner world but are good at cognitively reading the inner states of others. This ability can be applied to coerce or manipulate others. Although they are experts at understanding others cognitively, they cannot generate how they would feel in other people’s situations. This is connected to their impaired ability to read, recognize, and understand emotions (Bateman and Fonagy, 2016, p. 378). In particular, individuals with ASPD show deficits in the recognition of fearful emotions in others (Marsh and Blair, 2008) and they have a tendency to treat other people as objects instead of human beings (Bateman and Fonagy, 2016, p. 68). These characteristics make them more prone to engage in antisocial behavior, such as violent and aggressive acts toward other people.

In MBT, the clinician strives to develop a therapeutic process where focus is on mentalizing. This means that the patient becomes more aware of his/her thoughts and feelings toward themselves or others; how that affects responses to others; and whether “errors” in understanding themselves and others may lead to certain actions (Bateman and Fonagy, 2016, p. 147). However, MBT is primarily concerned with the process of mentalizing, and not the accuracy of one’s interpretations of mental states. The overall goals of MBT-ASPD are to stimulate robust mentalization and reduce aggression as the main pathway for expressing problematic internal states. Interventions in MBT-ASPD are especially concerned about the specific mentalizing deficits that individuals with ASPD exhibit. The clinician works with the patient to increase the understanding of self; build up a capacity to facilitate an understanding of other people’s feelings; and identify the sensitivity to hierarchical and rigid aspects of relationships (Bateman and Fonagy, 2016, p. 380).

A strength of MBT-ASPD is that it is aimed at treating personality difficulties, not comorbid disorders which have been found to be the case for previous attempts to treat the disorder (e.g., Gibbon et al., 2020). Even though MBT-ASPD has been specifically designed for ASPD, since mentalizing is a universal human capacity there is reason to believe that symptoms of comorbid disorders, such as SUD, anxiety, and depression, also can be reduced when enhancing mentalizing. This has for example been found for MBT for BPD. In a systematic review assessing the evidence for MBT’s effectiveness for BPD and comorbid disorders, they found a statistically significant reduction in depressive and anxiety symptoms (Vogt and Norman, 2019). Similarly, reduction of SUD was found in a feasibility study on MBT for BPD/SUD (Morken et al., 2017b).

In the Cochrane Review (2020) two ongoing RCTs that were assessing the effectiveness of mentalization-based treatment (MBT) in the treatment of ASPD, were identified. One of the RCTs is comparing probation as usual (PAU) supplemented with MBT to standard PAU for male offenders in community settings (Fonagy et al., 2020). The other RCT is comparing MBT- Introductory group (MBT-I) with TAU for male prisoners with BPD and/or ASPD (Gibbon et al., 2020). Although the studies were not included in the review, these trials could represent different interventional approaches to the treatment of ASPD in future updates of the review (Gibbon et al., 2020).

MBT- ASPD seems to have potential in treating ASPD although the evidence base is quite limited. If this is true one should assume that MBT-ASPD has the potential to reduce the therapeutic pessimism prevailing this patient group. We suspect that there are important components of this tailored treatment that ensure that these obstacles are overrun and that this could also be true for other specialized treatments for ASPD.

## Discussion

Confusion about the term ASPD, treatment rejecting behavior, refusing ASPD patients from treatment, and insufficient management of negative countertransference reactions in clinicians seem to be important variables for therapeutic pessimism among health professionals. To facilitate therapeutic optimism and more effective treatment outcomes, MBT-ASPD should have theoretical and practical solutions to solve these challenges.

### Psychopathy as an exclusion criterion

As stated earlier, individuals with ASPD and individuals with psychopathy could constitute two qualitatively different groups, and using the terms interchangeably may prevent effective treatment for both disorders (Ogloff et al., 2016). Within MBT-ASPD the two groups are viewed as qualitatively different; therefore, clinicians are explicitly advised to exclude people with personality features primarily associated with psychopathy (Coid and Ullrich, 2010; Bateman and Fonagy, 2016, p. 69; Bo and Kongerslev, 2016). This is primarily because the theoretical model in MBT is believed to fit psychopathy badly (Poythress et al., 2010). Additionally, the mentalizing abilities in individuals with ASPD with and without psychopathy seem to differ (Dolan and Fullam, 2004). De Visser et al. (2023) points out that for individuals with psychopathy there is a lack of motivation to mentalize others, while for ASPD patients without psychopathy there is a lack of ability to mentalize others. It is probably difficult to engage psychopathic individuals in authentic curious investigation of other people’s mind, MBT would therefore be a bad fit.

Dolan and Fullam (2004) conducted an RCT where they investigated theory of mind (ToM) and mentalizing ability in 89 criminal individuals with ASPD, with (n = 30) or without (*n* = 59) psychopathy, and 20 healthy individuals. The researchers identified several differences between individuals with ASPD with or without psychopathy. The individuals with psychopathic traits, on the other hand, did not express any marked difficulties in reading basic or complex emotions from facial expressions. They even demonstrated better skills than the control group in some areas of complex emotional recognition (Dolan and Fullam, 2004). This substantiates the assumption that individuals with ASPD and individuals with psychopathy constitute two different groups. As for mentalizing capacities, both groups performed worse than their healthy counterparts on subtle tests of mentalizing ability. However, individuals with ASPD without psychopathy and with neurotic features seem to have more impaired mentalizing abilities than individuals with psychopathy and low levels of anxiousness. Thus, the lack of motivation to engage with others, is probably a prognostically negative factor that should be considered when providing psychotherapy for ASPD with psychopathy.

For individuals with psychopathic traits, their main difficulties are not primarily related to mentalizing deficits, but rather to their lack of concern for other people (Dolan and Fullam, 2004; De Visser et al., 2023). For that reason, MBT may not be as effective for this group. Consequently, it is important with a screening of psychopathic traits in the introductory phase, before deciding whether a patient is eligible for MBT-ASPD. However, it is important to be cautious about not expanding this group too widely. Some traits, such as callousness may be mistaken as psychopathic traits, when in fact they are expressions of anxiety and insecure attachment in the individual (Bateman and Fonagy, 2016, p. 69). It is, nevertheless, important to note that although clinicians are advised to exclude people with personality features primarily associated with psychopathy in MBT programs, this does not mean that individuals with psychopathy cannot profit from elements in MBT. Mentalization has been found to have a mediating effect on aggression (Schwarzer et al., 2021), which is a common characteristic among individuals with a psychopathic personality (Dolan and Doyle, 2000; Monahan et al., 2001; Hare and Neumann, 2008; Leistico et al., 2008). In the MBT trial on conduct disorder in Germany, psychopathy is not an exclusion criterium (Taubner et al., 2021). Perhaps focusing on mentalization can be beneficial when treating individuals with psychopathy too.

### Reducing treatment-rejecting behavior

Individuals with ASPD are usually treatment-rejecting rather than treatment-seeking (Tyrer et al., 2003). Therefore, common challenges for therapists are to engage individuals with ASPD in therapy and prevent drop-out. These are challenges for MBT therapists as well. Thus, MBT-ASPD facilitates an extended period of engagement and motivational work before the formal treatment program starts. Additionally, attendance can be encouraged by calling the individuals 24 h before each group session and/or after missed sessions (Bateman and Fonagy, 2016, p. 395). According to MBT, motivation will not be enhanced if the clinician tries to motivate the participant to attend group sessions for the sake of the group members. Therefore, MBT suggests that it is better to tell the participants that the clinician’s work is to do whatever they can to work on their problems from a practical and psychological point of view, but that it is only achievable through regular attendance. Together the clinician and patient write down an agreed hierarchy of therapy-interfering behaviors or problems that are likely to happen (Bateman and Fonagy, 2016, p. 381).

There are several characteristics of individuals with ASPD that could impede the development of a therapeutic relationship. The sensitivity to power and hierarchy in relationships is one such example. In MBT-I, this issue is introduced and discussed. In the group sessions, the therapist needs to tolerate some resistance before these attitudes can be safely explored and understood within the group (McGauley et al., 2011). Many individuals with ASPD come from a criminal subculture with a luxurious lifestyle, are highly respected, and are at the top of their hierarchy. It is reasonable to think that going from a high-status lifestyle to therapy, which is associated with low status and weakness, leaves the individual vulnerable and lowers the threshold for impaired mentalization. Further, individuals with ASPD typically have resistance to authorities. Therefore, the MBT therapist should exhibit an authoritative style rather than an authoritarian one (Bateman and Fonagy, 2016, p. 395). Research supports this, as it makes it more likely to reduce risk and improve the therapeutic relationship (Meloy and Yakeley, 2014, p. 1,018). It is essential that the clinician and the participants develop a collaborative therapeutic relationship. Moreover, the clinician validates the participants’ perspectives but also facilitates enhanced mentalizing by trying to get the participants to look at other perspectives they have not yet considered. The clinician must by all means avoid ending up in an argument with the participants (Bateman and Fonagy, 2016, p. 392). Instead, the therapist should keep a not-knowing stance, being an active and curious questioning clinician (Bateman and Fonagy, 2016, p. 186).

Although MBT-ASPD appears to reduce therapy-interfering behaviors and prevent treatment drop-out, it is ultimately up to the individuals with ASPD whether they want treatment. Consequently, for them to want to engage and remain in therapy, it is important that they experience the treatment as meaningful. In 2019, Thomas and Jenkins conducted the first qualitative study on service users’ experiences with MBT-ASPD. All participants (*n* = 6) were offenders diagnosed with ASPD, participating in a 12-month community MBT program. In short, the findings showed that the participants valued the voluntary aspect of MBT and the combination of individual and group sessions. Furthermore, they appreciated the curiosity and the non-judgmental style in the group, and that the group helped them to deal with emotions and relationships in a safe and trustworthy environment.

The participants experienced enhanced mentalizing capacities, such as better listening skills and tolerance for perspectives different from their own. Lastly, many noticed a change in their relationships and their view of authorities. This was recognized by a feeling of responsibility for their own actions and a less rigid attitude of blaming others (Thomas and Jenkins, 2019).

This study gives important insight into how individuals with ASPD experience MBT. The findings illustrate how therapists were able to engage the participants in treatment. In particular, the voluntary aspect of MBT appears to be especially important. This has been highlighted in several studies (Ware et al., 2016; Thomas and Jenkins, 2019) and is probably different from what many with ASPD are used to. For example, Ware et al. (2016) displayed how the participants valued having ownership of their attendance and what was discussed in the group, and that this contributed to their regular attendance in the group. Additionally, another important facet to consider is MBT’s contribution to the so-called epistemic trust toward the clinician. Epistemic trust is defined as “the willingness to accept new information from another person as trustworthy, generalizable, and relevant” (Schröder-Pfeifer et al., 2018). Thomas and Jenkins (2019) argued that their participants showed epistemic trust because they gradually became more flexible in their thinking and reported feelings of belonging.

However, Thomas and Jenkins (2019) also identified some challenges. The participants acknowledged that the process of mentalizing is difficult and requires a lot of effort. Additionally, some reported that in the early stages of the program, the “curious stance” of the clinician was not perceived as curiosity, but rather as an implied ulterior motive causing distress. Consequently, they initially doubted the program. It is unknown whether this perception is caused by the individual clinician with an exaggerated “not- knowing” stance (Inderhaug, 2013), or whether it is caused by the MBT framework itself. It is plausible that this experience is common for many individuals with ASPD. Therefore, in order to develop epistemic trust, the initial challenges of perceiving the clinician’s “curious stance” as genuine need to be overcome. Additionally, the study included few participants, which limits the generalizability of the findings. Besides, 50% of the participants were recalled to prison either during the program or after completion (Thomas and Jenkins, 2019). One may therefore question whether MBT actually can contribute to progress and change.

However, the themes are similar to findings in other studies investigating service users’ experiences of MBT. This includes positive experiences in attachment from BPD patients (Dyson and Brown, 2016), participants with BPD and/or ASPD in a high-secure forensic hospital experiencing that MBT improved their capacity to manage their own emotions and behavior (Ware et al., 2016), and BPD patients who reported that MBT interventions contributed to a degree of changing perspectives (Lonargáin et al., 2017; Morken et al., 2017a).

### Accepted rather than denied into treatment

The findings from various studies show that lack of experience, knowledge, and perceived behavioral control are components that contribute to negative attitudes, therapeutic pessimism, and low willingness to work with individuals with ASPD among health professionals (Eren and Şahin, 2016; van Dam et al., 2022). Contrarily, education, expertise, and clinical supervision seem to contribute to more positive attitudes around treatment opportunities concerning ASPD. What is MBT’s contribution to this?

Adjusting the original MBT approach for individuals with ASPD to weekly group sessions and monthly individual sessions; assigning clinicians the dual role of being individual and group therapists; and putting more emphasis to uphold frames and structure, would fit individuals with ASPD to a greater extent (Bateman and Fonagy, 2016). MBT is, compared to TAU, tailored to treat the actual difficulties that individuals with ASPD are struggling with, for example mentalizing the other to improve empathy, managing and regulating difficult feelings, and the focus on attachment and hierarchical relationships. Additionally, even though a dimensional view of personality disorders is taking place (e.g., ICD-11), MBT as a specified treatment manual could still be used. During the assessment interview, the clinician gathers information about the individual’s specific mentalizing deficits resulting in a mentalizing profile. The mentalizing profile shows the specific combinations of malfunction in the different aspects of mentalizing for the specific individual with ASPD. This combination of mentalizing deficits provides crucial information about what interventions the individual is likely to benefit from (Bateman and Fonagy, 2016, p. 8). For that reason, specific mentalizing deficits are more indicative of treatment than diagnosis.

MBT also relies heavily on supervision (Morken et al., 2022), and the MBT therapists are supervised individually and in teams by a senior member of the team (Bateman and Fonagy, 2016, p. 436). The goal is to maintain a mentalizing stance, recognized by a not- knowing, non-judgmental, curious, and open attitude (Bateman and Fonagy, 2016, p. 186). This means that the team should act in the same way as they try to model to their patients. Besides, sessions are videotaped to ensure sufficient supervision and contribute to research (e.g., Morken et al., 2022). In sum, a clear treatment manual, supervision, and research are crucial parts of MBT. It is likely that this will improve experience, knowledge, and perceived behavioral control for clinicians in the treatment of ASPD.

A final critical aspect is the stigmatization and pessimism surrounding the disorder. In turn, this could lead to certain punitive attitudes. MBT could play an important role in reducing stigma and pessimism in several ways. The clinicians’ attitude in MBT recognized by a curious and non-judgmental attitude, signalizes an openness and desire to help and understand rather than a desire to punish and judge. Additionally, MBT’s understanding of ASPD as a developmental disorder rooted in insecure attachment could have important implications. First, this perspective can allow for early interventions in families at risk and children with conduct disorder. Second, it could inform treatment for adults with the diagnosis (Yakeley, 2014). In MBT theory, insecure attachment is not irreversible, an assumption that could institute hope in clinicians and patients.

It is, however, important to hold the individuals accountable for their actions. Some critics may argue that MBT focuses too much on compassion and understanding for individuals with ASPD. The view on treatment versus punishment is controversial. While punishment could have a deterrent effect, meaning that other people might stand off from similar behavior because they know the consequences of these actions (Galbiati and Drago, 2014), punitive measures may not always be the most effective way to address their behavior. Additionally, as a society, we have an ethical obligation and duty to provide care to those who are suffering from mental health disorders (National Institute for Health and Care Excellence, 2010).

Altogether, the combination of viewing ASPD as a developmental disorder and the general attitudes in MBT may change negative views on treatability, decrease stigmatization, and change punitive attitudes. Considering the individuals as being exposed to severe trauma and/or neglect may change the negative attitudes concerning that all individuals with ASPD only deserve punishment. It is reasonable to believe that this could reduce stigma among health professionals and facilitate therapeutic optimism.

### Managing countertransference

Studies indicate that it is the lack of sufficient management of countertransference reactions, not countertransference itself, that may have a therapy-inhibiting effect (Hayes et al., 2018). Therefore, it is interesting to take a closer look at how MBT specifically facilitates good management of countertransference reactions among therapists. Little research is done on therapist experiences with MBT-ASPD (Morken et al., 2022). In order to gain better insight into therapist experiences and perceived well-being of providing MBT-ASPD, Morken et al. (2022) conducted a qualitative study. The participants were experienced therapists participating in an ongoing pilot study examining the potential treatment outcomes of MBT for individuals with ASPD and comorbid substance use disorders. The researchers identified four main themes covering the therapists’ clinical experiences: (i) gaining safety by getting to know them better, (ii) gaining cooperation through clear boundaries and a non-judgmental stance, (iii) shifting inner boundaries, and (iv) timing interventions in a high-speed culture.

The researchers also proposed specific suggestions for how therapists providing MBT-ASPD can manage negative countertransference reactions based on their findings and relevant literature. First, the researchers emphasized the importance of clear procedures. This includes peer supervision and regular communication between the therapists about their reactions. The findings revealed that there were individual differences in how the therapists were affected by the countertransference reactions. Nevertheless, it is essential to create an open, supportive, and transparent mentalizing environment, both among clinicians and in therapy sessions (Morken et al., 2022). In MBT-ASPD the clinicians are encouraged to engage in the juxtaposition of their own mental states (Bateman et al., 2013). The juxtaposition of one’s own mental state refers to clearly and directly communicating to the patient what kinds of feelings different subjects evoke. This is especially important when difficult subjects are discussed by patients in a limitless way, in order to contrast their views and stimulate mentalization. Lastly, the researchers underlined the importance of strictly upholding the MBT-ASPD treatment format. To summarize, monitoring countertransference reactions and ensuring support to therapists providing treatment to individuals with ASPD, seem to be particularly important in order to manage negative countertransference reactions (Morken et al., 2022).

According to Yakeley and Williams (2014), having a focus on countertransference in therapy is among the things that characterize the most efficient treatments for individuals with ASPD. MBT stresses the importance of working with transference and countertransference because unresolved conflicts linked to these processes can be a therapy- inhibiting factor and undermine therapists’ and participants’ mentalizing abilities (Bateman et al., 2014, p. 24). Furthermore, engaging the patient in an exploratory and mentalizing therapeutic dialog is believed to be a crucial factor for good therapy outcomes (Karterud et al., 2020, p. 167). However, in MBT, therapists are not encouraged to engage in transference interpretation (Karterud et al., 2020, pp. 167, 173). Instead, they should mentalize the transference refers to the process where the therapist actively encourages the patient to focus and work on the therapeutic relationship here and now (Bateman and Fonagy, 2016, p. 275). The purpose of this is to orient the patient’s attention toward another person’s mind. This can facilitate mentalization and help them to contrast their perception of themselves with others’ perception of them, e.g., the therapist (Bateman and Fonagy, 2010; Karterud et al., 2020, p. 167). Therapists’ countertransference reactions partly affect their perception of their patients, and monitoring one’s countertransference reactions is therefore crucial when mentalizing the transference. According to Bateman et al. (2014, p. 72), good skills in mentalizing the transference include an “ability to monitor countertransference and to work to regain a reflective stance after an enactment.”

In addition to focusing on the relationship to the therapist, use of countertransference is essential in MBT. Therapists should implement their thoughts and feelings about the therapeutic relationship in ways that stimulate the therapeutic process. The therapist should also aim to maintain this process by creatively stimulating the patient’s curiosity and exploration. By using countertransference, the therapist can model authenticity and a willingness to explore one’s own feelings in an open and honest way (Karterud et al., 2020, p. 173). The therapist should also strive to demonstrate to the patient how one can regain one’s mentalization capacity after mentalization failure (Karterud et al., 2020, p. 175).

**Figure 2:**
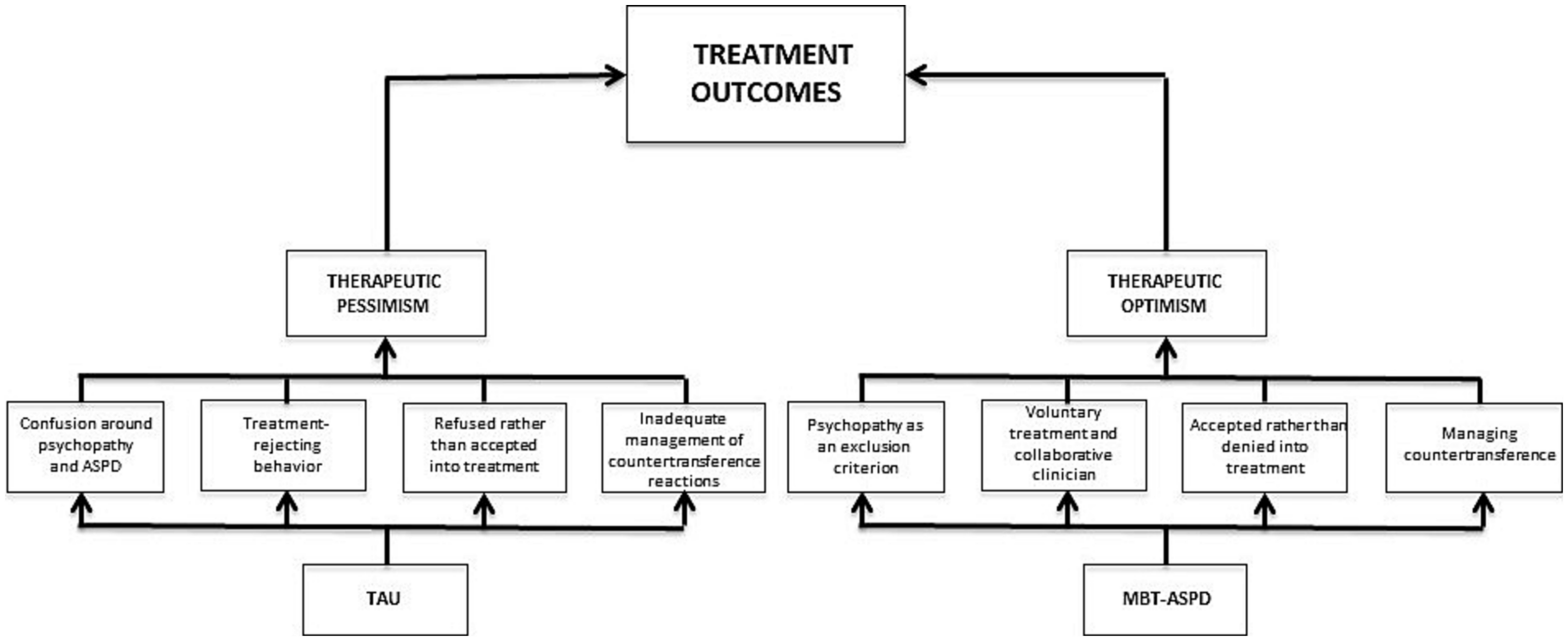
MBT-ASPD’s possible contributions to therapeutic optimism and more effective treatment outcomes for individuals with ASPD.

Overall, as Figure 2 demonstrates, several variables, including insufficient management of countertransference reactions, appear to contribute to the therapeutic pessimism toward individuals with ASPD. MBT has explicitly incorporated countertransference in the treatment. The fact that MBT openly uses countertransference, in sessions and through systematic supervision, seems to be particularly important for good management of negative countertransference reactions (Bateman et al., 2014, p. 22) and for reducing the risk of unproductive therapeutic work (Karterud et al., 2020, p. 229). This is thought to be a unique aspect of MBT-ASPD and could serve as a sufficient way to manage the negative countertransference reactions in clinicians when working with ASPD patients. MBT-ASPD targets the other variables contributing to therapeutic pessimism too. First, psychopathy is an exclusion criterion in MBT-ASPD, and this limits the confusion around ASPD and psychopathy. Second, MBT-ASPD is a voluntary treatment with a collaborative, authoritarian clinician, which facilitates a stronger working alliance and could serve as an important tool to reduce treatment-rejecting behavior. Third, the fact that MBT views ASPD as a developmental disorder rooted in insecure attachment, may reduce stigma and contribute to an acceptance rather than a refusal of individuals with ASPD in therapy. Besides, a clear treatment manual, supervision, and training can make up for the lack of experience, knowledge, and expertise that seems to hinder many health professionals from working with individuals with ASPD.

As shown in Figure 2, variables related to both therapeutic pessimism and therapeutic optimism could potentially affect treatment outcomes. The figure demonstrates MBT-ASPD’s contribution to a therapeutic willingness toward individuals with ASPD among health professionals. We hypothesize that MBT-ASPD may be an important contribution to increased therapeutic optimism and that this, in turn, can result in more effective treatment outcomes for individuals with ASPD.

## Conclusion

This review has delved into the therapeutic pessimism concerning ASPD among health professionals and suggested how MBT-ASPD can solve the different obstacles by (1) differentiating between psychopathy and antisocial personality disorder, (2) accept patients to treatment by providing a manual and systematic supervision, (3) explicitly focus on countertransference within the therapists and (4) emphasize voluntary treatment and collaboration with the patient (see Figure 2). It appears that specialized psychotherapies, for example, MBT-ASPD can be well-suited for the treatment of challenging patient groups, such as individuals with ASPD. This theoretical review illustrates the probable importance of specialized psychotherapies. TAU appears to fall short with regard to the treatment of ASPD, which indicates a need for a treatment specifically tailored for this group. It is important that the healthcare system takes different needs into account in order to achieve more effective treatment for challenging patient groups. Moreover, more effective treatment requires a change in attitudes among health professionals. Changes on how mental health systems implement and organize treatment deliverance is crucial when working with patient groups with high severity. Understanding ASPD as a developmental disorder seems to be an important component in order to decrease negative attitudes. Increased therapeutic optimism among health professionals could, in turn, indirectly affect the organization of the healthcare system and, thus, facilitate more effective treatment options for individuals with ASPD.

Although the effectiveness of MBT-ASPD is still uncertain, our findings indicate that there is ground for therapeutic optimism concerning ASPD. Newer perspectives on psychotherapeutic treatment of ASPD is encouraging and optimistic (for example; Van den Bosch et al., 2018; Bateman, 2022; De Visser et al., 2023).While awaiting an effective treatment of ASPD, we hope that this review can contribute to an alternated view on the treatment opportunities of ASPD, increase focus on structural components around psychotherapy and emphasize the societal and economic benefits of finding an effective psychological treatment. Additionally, we hope that this review can contribute to an alteration of punitive attitudes toward ASPD in society to foster therapeutic initiatives to better help an underserved patient group.

## Limitations

Although the theoretical assumptions in this review support that MBT can alleviate the negative impact of the therapeutic pessimism among health professionals toward individuals with ASPD, there are some limitations. There is limited evidence base on MBT-ASPD and several studies on MBT-ASPD have been conducted quite recently. Therefore, knowledge about the long-term consequences of the treatment is limited. Furthermore, studies on MBT-ASPD have few participants (e.g., McGauley et al., 2011). Small samples can affect the external validity of the findings negatively (Price and Murnan, 2004). As with all diagnoses, individuals with ASPD are a heterogeneous group. Thus, the participants in the studies we have included do not necessarily constitute a representative group, and this can limit the generalizability of their findings (American Psychological Association, n.d.-b).

Additionally, since RCTs on MBT-ASPD are yet to be published, we have not included any RCTs in our review, nor have we focused on other forms of treatment for ASPD. It is potentially problematic that we did not differentiate between treatment modalities such as forensic mental health services, addiction health services and psychiatric health services. However, our purpose was to investigate the relationship, not the causality, between therapeutic pessimism toward individuals with ASPD and MBT. As RCTs are considered the gold standard for effectiveness research (Hariton and Locascio, 2018), we hope future research takes this into consideration as it would be interesting to measure the effectiveness of MBT-ASPD compared to other forms of treatment. We look forward to the findings from the ongoing RCTs on MBT-ASPD (e.g., Fonagy et al., 2020), as well as studies investigating other treatments specifically targeted for ASPD.

### Future directions

Certain limitations of the included studies in this review could be addressed in future research. This review focuses primarily on MBT for ASPD. In order to increase the likelihood of providing the best approaches for patients with ASPD, all effective methods should be taken into consideration. Furthermore, MBT ASPD has primarily been tested on male patients and we therefore do not know how this relates to female ASPD.

One attempt at a more holistic approach to ASPD could be that future research investigates the contributions of family involvement. National Institute for Health and Care Excellence (2010) guidelines underline the importance of including family and caretakers in the treatment. For BPD, MBT has implemented a family and carers training and support program (FACTS), a program implemented to teach families about BPD and learn about basic skills on how to manage common problems (Bateman and Fonagy, 2016, p. 416). One RCT found that this implementation reduced adverse incidents within the families (Bateman et al., 2019). This inclusion of the environment around the individual with the personality disorder could be important for obtaining comparative information and improving family relationships. Based on what we can find from the existing literature, no such implementation has been created for ASPD.

Future research should also shed light on a systemic, interdisciplinary approach to ASPD. A significant portion of the available research on ASPD is conducted in forensic settings, as many individuals with ASPD are involved in the criminal justice system. The upcoming RCT on MBT is also investigating offending male adults in community probation (Fonagy et al., 2020). However, as this review has demonstrated, not all individuals with ASPD commit crimes (National Institute for Health and Care Excellence, 2015). It would be interesting if future research could compare the differences and similarities between what is done in forensic and regular mental health settings and what the two could learn from one another. One promising study from Van den Bosch et al. (2018) has demonstrated how managerial conditions are of great importance in the treatment delivery for patients with ASPD, we speculate that different areas within our health services differ in these conditions and therefore matter for the outcome of the treatment. Studies looking closer at managerial and systemic conditions would be exciting.

Lastly, future research should look into preventive measures and treatment options for individuals at risk of developing ASPD. It is known that a substantial portion of children and youths diagnosed with conduct disorder will develop ASPD in adulthood (National Institute for Health and Care Excellence, 2010). National Institute for Health and Care Excellence (2010) focuses on recommendations for early interventions for children diagnosed with, or at risk of developing, conduct disorder. Current research now supports the idea that some variants of ASPD can be understood as a developmental disorder rooted in insecure attachment (Yakeley and Williams, 2014; De Visser et al., 2023). In terms of future research, it would be useful to extend the current findings by examining what implications this could have for the prevention of ASPD and early intervention for individuals with conduct disorder. A recently published feasibility study in MBT for conduct disorder has demonstrated significant clinical changes for youth with conduct disorder, in MB-CD psychopathy and antisocial PD traits are both included (Hauschild et al., 2023) which provides therapeutic optimism for this patient group. Different clinical pathways were recommended by De Visser et al. (2023) with emphasis on trauma focused interventions within psychotherapeutic settings for those with attachment related disturbances and ASPD. MBT should also be promising for managing those with attachment related distorted social cognition.

## Author contributions

EF: Conceptualization, Formal analysis, Writing – original draft, Writing – review & editing. ML: Conceptualization, Formal analysis, Writing – original draft, Writing – review & editing. KM: Conceptualization, Supervision, Writing – review & editing.
